# Ginsenoside Rg3 induces apoptosis and inhibits proliferation by down-regulating TIGAR in rats with gastric precancerous lesions

**DOI:** 10.1186/s12906-022-03669-z

**Published:** 2022-07-15

**Authors:** Shangbin Lv, Xiaodong Chen, Yu Chen, Daoyin Gong, Gang Mao, Caifei Shen, Ting Xia, Jing Cheng, Zhaoliang Luo, Yu Cheng, Weihong Li, Jinhao Zeng

**Affiliations:** 1 Basic Medical College, Chengdu University of Traditional Chinese Medicine, Chengdu, China; 2 Department of Gastrointestinal Surgery, Sichuan Cancer Hospital, School of Medicine, University of Electronic Science and Technology of China, Chengdu, China; 3 Hospital of Chengdu University of Traditional Chinese Medicine, Chengdu, China; 4 Digestive Endoscopy Center, Hospital of Chengdu University of Traditional Chinese Medicine, Chengdu, China; 5 Chengdu University of Traditional Chinese Medicine, Chengdu, China; 6 Sichuan University West China Hospital Ganzi Hospital, Ganzi, China; 7 Department of Chinese Internal Medicine, Hospital of Chengdu University of Traditional Chinese Medicine, Chengdu, China; 8 TCM Regulating Metabolic Diseases Key Laboratory of Sichuan Province, Hospital of Chengdu University of Traditional Chinese Medicine, Chengdu, China

**Keywords:** Ginsenoside Rg3, Gastric precancerous lesions, Apoptosis, TP53-induced glycolysis and apoptosis regulator, Reactive oxygen species

## Abstract

**Background:**

Ginsenoside Rg3 (GRg3) is one of the main active ingredients in Chinese ginseng extract and has various biological effects, such as immune-enhancing, antitumour, antiangiogenic, immunomodulatory and anti-inflammatory effects. This study aimed to investigate the therapeutic effect of GRg3 on gastric precancerous lesion (GPL) induced by N-methyl-N′-nitro-N-nitrosoguanidine (MNNG) and the potential mechanism of action.

**Methods:**

The MNNG–ammonia composite modelling method was used to establish a rat model of GPL. Histopathological changes in the rat gastric mucosa were observed by pathological analysis using haematoxylin–eosin staining to assess the success rate of the composite modelling method. Alcian blue–periodic acid Schiff staining was used to observe intestinal metaplasia in the rat gastric mucosa. Apoptosis was detected in rat gastric mucosal cells by terminal deoxynucleotidyl transferase-mediated dUTP nick end labelling staining. The production level of reactive oxygen species (ROS) was determined by the dihydroethidium fluorescent probe method, and that of TP53-induced glycolysis and apoptosis regulator (TIGAR) protein was determined by immunohistochemical staining and western blotting. The production levels of nicotinamide adenine dinucleotide phosphate (NADP) and glucose-6-phosphate dehydrogenase (G6PDH) were determined by an enzyme-linked immunosorbent assay, and that of glutathione (GSH) was determined by microanalysis.

**Results:**

GRg3 significantly alleviated the structural disorganization and cellular heteromorphism in the form of epithelial glands in the gastric mucosa of rats with GPL and retarded the progression of the disease. Overexpression of TIGAR and overproduction of NADP, GSH and G6PDH occurred in the gastric mucosal epithelium of rats with GPL, which in turn led to an increase in the ROS concentration. After treatment with GRg3, the expression of TIGAR and production of NADP, GSH G6PDH decreased, causing a further increase in the concentration of ROS in the gastric mucosal epithelium, which in turn induced apoptosis and played a role in inhibiting the abnormal proliferation and differentiation of gastric mucosal epithelial cells.

**Conclusion:**

Grg3 can induce apoptosis and inhibit cell proliferation in MNNG-induced GPL rats. The mechanism may be related to down-regulating the expression levels of TIGAR and production levels of GSH, NADP and G6PD, and up-regulating the concentration of ROS.

## Introduction

Gastric precancerous lesion (GPL) is usually classed as intestinal metaplasia (IM) or gastric epithelial dysplasia (DYS). GPL is closely associated with the pathogenesis of gastric cancer (GC). The latest data show that GC is the fifth most common cancer worldwide and the fourth leading cause of death among cancer patients worldwide [1]. Because there is a direct link between the development of GC and GPL, preventing the progression of GPL to GC is the key to reducing the incidence of GC [2]. Modern medicine does not have specific drugs for GPL, and endoscopic mucosal resection is only performed when a patient is diagnosed with severe gastroesophageal reflux disease and definite intramucosal cancer. However, most patients with GPL cannot undergo endoscopic resection, and modern medicine thus lacks effective interventions for GPL [3]. Therefore, the search for simple and effective alternative treatments has always been an important topic of research.

Ginsenoside Rg3 (GRg3) is one of the main active ingredients extracted from *panax ginseng* and has a wide range of pharmacological activities, including immune-enhancing, antitumour, antiangiogenic, immunomodulatory, anti-inflammatory and antioxidant effects [4–8]. GRg3 can also be used as an adjuvant for traditional cancer treatments to reduce their toxicity and increase their efficiency via its synergistic action [9].

Moreover, GRg3 has been shown to significantly inhibit tumour growth in different cancer models [10, 11]. Recent studies have shown that its activity is mainly associated with the induction of apoptosis, inhibition of proliferation and angiogenesis, regulation of immunity and inhibition of multidrug resistance, which increases chemosensitivity [12]. GRg3 has been found to be effective against GC in in vivo and in vitro studies and might be a promising agent for the treatment of GC. In vitro tests showed that GRg3 inhibited the activity of SGC-7901 cells by regulating the phosphatase and tensin homologue/p-phosphoinositide 3-kinase (PI3K)/Akt pathway [13]. Other tests showed that GRg3 inhibited the expression of hypoxia-inducible factor-1α and vascular endothelial growth factor in BGC823 human GC cells and thereby inhibited metastasis in GC [14]. GRg3 also inhibited fucosyltransferase-4-induced apoptosis in gastric mucosal cells by upregulating Sp1 and downregulating heat shock factor-1 [15]. Previous animal experiments found that GRg3 inhibits angiogenesis in gastric precancerous lesions through downregulation of Glut1 and Glut4 [16]. In order to further study the mechanism of Grg3 in the treatment of GPL, we used a rat model of GPL to investigate the interventional effects and potential mechanisms of GRg3 on the gastric mucosa of rats with N-methyl-N′-nitro-N-nitrosoguanidine (MNNG)-induced GPL.

It is well known that tumours grow because of uncontrolled cell proliferation [17] and that reactive oxygen species (ROS) are closely associated with the proliferation of tumour cells. When the ROS concentration is maintained at a fairly high level, cells are in a state of oxidative stress that cannot be alleviated by antioxidants. This can damage intracellular DNA, proteins and organelles and thus lead to lethal cell damage and trigger apoptosis [18]. However, if the ROS concentration is increased to an appropriate, nonlethal level, cells will instead gradually adapt. This creates a new redox balance and enables abnormal cell proliferation, which is associated with various processes such as tumour cell survival and proliferation, angiogenesis and metastasis [19, 20]. In rapidly proliferating cells, fluctuations in the production of both glutathione (GSH), which protects cells and biological macromolecules such as proteins from damage due to ROS, and nicotinamide adenine dinucleotide phosphate (NADP), which maintains GSH in its reduced state, occur as a result of an increase in the production of ROS [21]. Therefore, ROS have become an important subject of research from the perspective of cellular redox homoeostasis. TP53-induced glycolysis and apoptosis regulator (TIGAR) is a protein that regulates glucose-6-phosphate dehydrogenase (G6PDH), NADP and GSH, and an increase in its production leads to abnormal activation of G6PDH, NADP and GSH [22], of which the intracellular concentrations are thus maintained at high levels. This makes the antioxidant system in cells relatively stable, and ROS concentrations can be maintained at relatively high levels that enable rapid cell proliferation without triggering apoptosis [23, 24]. In GC, after knockdown of TIGAR cells would be unable to scavenge ROS because insufficient amounts of NADP, G6PDH and GSH were synthesized, and cells would be in a state of oxidative stress that activated the apoptosis pathway and inhibited excessive proliferation of GC cells [25, 26]. At present, however, there have been few studies of TIGAR in the GPL stage.

In this study, we investigated whether GRg3 can affect apoptosis of gastric mucosal cells by regulating TIGAR, GSH, G6PDH and NADP. In order to elucidate the potential mechanism of the intervention of GRg3 in GPL, we found evidence that GRg3 can play a role in promoting repair of the gastric mucosa and retarding the progression of the disease by triggering apoptosis.

## Materials and methods

### Animals

Seventy-four male Sprague-Dawley rats with weights of 190 ± 10 g and ages of 6–7 weeks were provided by Chengdu Dashuo Biotechnology Co., Ltd. and were housed in a laboratory at Chengdu University of Traditional Chinese Medicine on a 12 h/12 h light/dark cycle. The room temperature was maintained at 20 ± 2 °C, and the relative humidity was maintained at 50–70%. The animal experiments in this study were approved by the Experimental Animal Ethics Committee of Chengdu University of Traditional Chinese Medicine (audit filing number: 2019–17).

### Clinical sample

A total of 69 GPL samples and 52 normal samples were collected from patients who underwent gastroscopy at the Department of Gastroenterology, Hospital of Chengdu University of Traditional Chinese Medicine between January 2018 and May 2021. The paraffin embedded tissue samples were stored at room temperature. The procedures have been approved by the Medical Ethics Committee of Hospital of Chengdu University of Traditional Chinese Medicine (approval no: 2018KL-023). Signed informed consent forms were obtained from all participants.

### Establishment and treatment of rat model of GPL

**Fig. 1:**
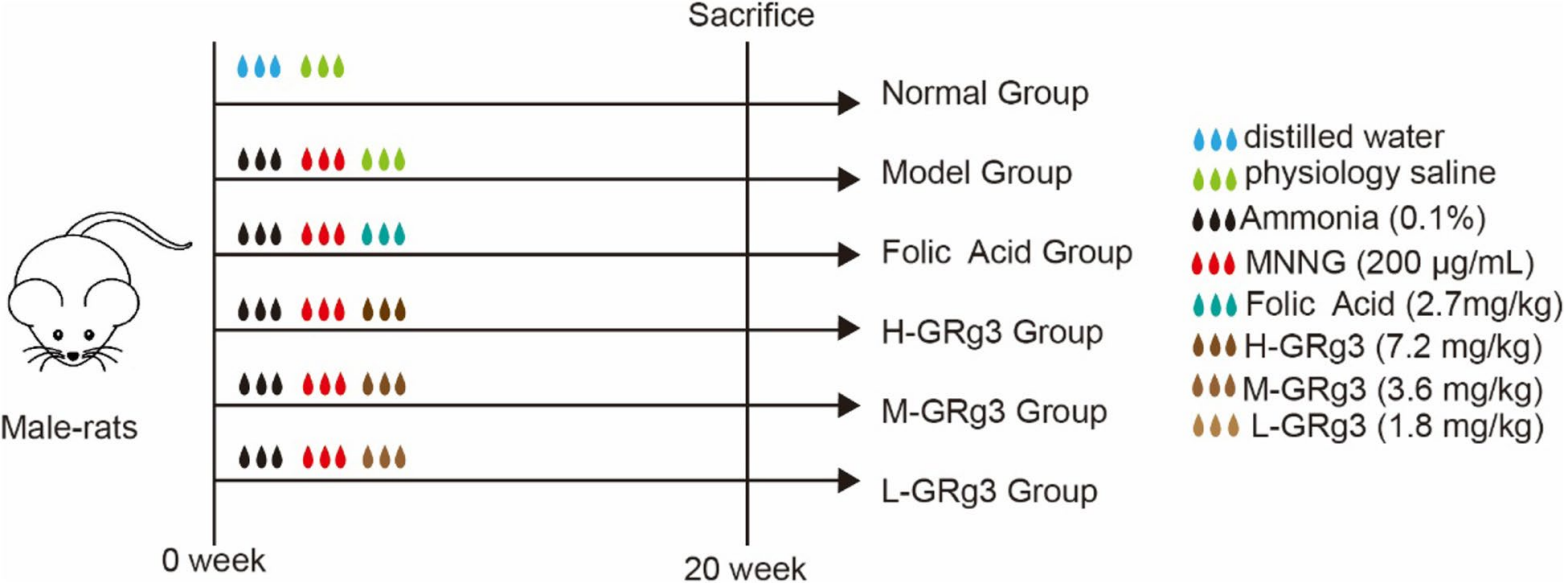
Scheme of the experimental design (*n* = 12 rats per group)

A rat model of GPL was established by the MNNG–ammonia composite modelling method. MNNG was purchased from Tokyo Chemical Industry Company, Japan (item number: CFFC-M0527-5G). The modelling process is shown in Fig. 1. First, the 74 rats were divided into normal (*n* = 12) and model (*n* = 62) groups using the digital random table method. The rats in the normal group were housed routinely, and the rats in the model group were given an ammonia solution with a concentration of 0.1% and were allowed to drink freely. Ammonia was purchased from China Sichuan Kebei Biological Company Limited (item number: GB/T631–2007). The rats in the model group received a 200 μg mL^− 1^ MNNG solution by gavage and were fasted twice a week for 24 h each time without drinking [27]. At the end of week 8, two animals were randomly selected from the model group, from which the gastric mucosa at the gastric body–sinus junction was removed and stained with haematoxylin and eosin (H&E) to confirm the presence of early GPL in the form of mucosal intestinal epithelial metaplasia under light microscopy. The rats in the model group were then randomly divided into a model group, a folic acid group and high-, medium- and low-dose GRg3 groups, each of which comprised 12 rats. All groups except the normal group continued to receive the 0.1% ammonia solution ad libitum and the 200 μg mL^− 1^ MNNG solution by gavage. Gavage treatment was started in week 9. The folic acid group received 2.7 mg kg^− 1^ day^− 1^ folic acid. Folic acid tablets were obtained from Changzhou Pharmaceutical Factory Co., Ltd. (lot number: H32023302). The high-, medium- and low-dose GRg3 groups received 7.2 mg kg^− 1^, 3.6 mg kg^− 1^ and 1.8 mg kg^− 1^ GRg3, respectively. GRg3 was supplied by Hefei Bomei Biotechnology Co., Ltd. (item number: BZP0239). The normal and model groups received 1 mL/100 g saline by gavage. The rats were treated by gavage for 12 weeks.

### Pathological analysis

After being fasted for 12 h, the animals were euthanized with sodium pentobarbital (2 mL g^− 1^). The stomach was immediately removed, cut along the greater curvature and fixed in 4% paraformaldehyde fixative. Each sample was then embedded in paraffin and serially sectioned to a thickness of 3 μm. The sections were stained with H&E to observe histopathological changes in the gastric mucosa. In addition, the type of IM was determined using Alcian blue–periodic acid Schiff (AB-PAS) staining. We scored HE staining and AB-PAS staining as follows: three visual fields were randomly selected under 200x light microscope to judge the pathological changes of DYS and IM in gastric mucosa of rats, according to the severity of the lesions (0, no histopathology lesion; 1, mild histopathology lesion; 2, moderate histopathology lesion; 3, severe histopathology lesion).

### Terminal deoxynucleotidyl transferase dUTP nick end labelling staining

TUNEL apoptosis assay was carried out according to the manufacturer’s instructions (Servicebio, No. G1507). Four micrometre-thick sections, sliced from paraffin-embedded gastric tissues, were routinely dewaxed and rehydrated. The sections were incubated with proteinase K working solution at 37 °C for 20 min, followed by incubation with labeling buffer containing Terminal deoxynucleotidyl Transferase (TdT) and digoxin labeled deoxyuridine triphosphate (dUTP). Next, samples were exposed to Reagent Streptavidin-HRP and TBST. Finally, staining with 3, 3′-diaminobenzidine (DAB) solution (Servicebio, No. G1212) and counterstaining with hematoxylin. Apoptotic cells were shown as nuclear staining. Counting for apoptotic cells was performed using light microscopy with magnification of × 400. Three randomly selected fields were captured for each section, in which apoptosis index was calculated.

### Dihydroethidium fluorescent probe method

Frozen sections were warmed to room temperature while the humidity was controlled and were then dried. An autofluorescence quencher (Servicebio, No. G1221) was added to the sections for 5 minutes, followed by a 10-minute rinse with tap water. The DHE (Sigma, D7008) was added dropwise to the sections, which were then incubated at 37 °C for 30 min. The sections were placed in PBS (pH 7.4), shaken and washed three times for 5 min each time on a decolorizing shaker. The sections were shaken, dried and sealed with an anti-fluorescence quenching sealer (Servicebio, No. G1401). The sections were observed under a fluorescence microscope, and images were collected with red fluorescence regarded as positive staining. The average absorbance values were calculated using ImageJ software.

### Western blotting

Gastric mucosal tissues were homogenized and lysed in RIPA buffer(Servicebio, NO. G2002) containing a protease inhibitor cocktail, and then the lysates were centrifuged at 12000 rpm for 10 min at 4 °C. The protein concentration was determined using a commercial BCA kit (Servicebio, NO.G2026-1000 T). Proteins were separated by SDS-PAGE on 8–10% gels and transferred onto PVDF membranes The membranes were decolorized at room temperature on a shaker with 5% skimmed milk in a 0.05% TBST preparation and were then incubated with a diluted primary antibody (anti-TIGAR antibody, Abcam, ab189164; anti-β-actin antibody; Abcam, ab8227) at 4 °C overnight. A secondary antibody (HRP-labelled goat anti-rabbit; Servicebio, No. GB23303) was then added at room temperature, and the mixture was held for 1 h. The protein expression was assessed using an ultra-enhanced chemiluminescence reagent (K003; Affinity Biosciences, Cincinnati, OH, USA). β-actin was used as an internal control, and the optical density (OD) of each band was determined using ImageJ software.

### Biological data mining methodology

Clinical data on GC and data on TIGAR expression were downloaded from the Cancer Genome Atlas (TCGA) database and included data for 32 normal samples and 375 GC samples. To investigate the biological processes involved in low and high expression of TIGAR in GC, gene set enrichment analysis (GSEA) was performed on the transcriptomics data from the TCGA on GC, which included data on 19,645 genes and 375 samples. The median value for the expression of TIGAR was used to classify these 375 samples into high- and low-expression groups, and GSEA software was used for analysis.

### Immunohistochemical staining

Gastric tissue was fixed in 4% paraformaldehyde for 24 h at room temperature, embedded in paraffin and cut into 4 μm-thick sections. The sections were dewaxed, dehydrated and treated with sodium citrate buffer for antigen repair. The sections were then immersed in a 3% H2O2/ethanol solution to inhibit endogenous peroxidases and were then blocked with 3% bovine serum albumin. The sections were incubated overnight at 4 °C with the primary antibody (anti-TIGAR antibody; Abcam, ab189164, anti-PCNA antibody; Abcam, ab189164). The samples were then exposed to secondary antibodies (HRP-labelled goat anti-rabbit Servicebio, No. GB23303) at room temperature for 20 min. Samples were then stained with DAB solution for 1 min and counterstained with hematoxylin for 20 sec at room temperature. Each slide was examined microscopically, and three visual fields were randomly selected. TIGAR and PCNA were observed under 400× optical microscope. Brownish-yellow particles were regarded as indicating positive expression.

To conduct IHC analysis of human gastric mucosa samples, 4-μm-thick sections were cut from paraffin-embedded samples. The sections were dewaxed, dehydrated and treated with sodium citrate buffer for antigen repair. The sections were then immersed in a 3% H2O2/ethanol solution to inhibit endogenous peroxidases and were then blocked with 3% bovine serum albumin. The sections were incubated overnight at 4 °C with the primary antibody (anti-TIGAR antibody; Abcam, ab189164). After the primary antibody was incubated, the slices were incubated with HRP/Fab secondary antibody (Servicebio, No. GB23303) at room temperature for 20 min. Tissue sections were stained with DAB solution for 5 min at room temperature and then restained with hematoxylin for 20 s. Each slide was analyzed by light microscope. The magnification used was 200x and 400x.

We also determined the expression level of TIGAR in human gastric mucosa using the immunoreactivity score (IRS) in the following way: we invited two pathologists to assess the intensity of immunohistochemical (IHC) staining of TIGAR in the human gastric mucosa in a randomized manner using magnifications of 400× without clinicopathological data. The percentage of positive cells was calculated by IPP software, and the IRS was calculated for the semi-quantitative determination of human TIGAR. The IRS was determined from the sum of the percentage of positive cells (0 points, 0–5%; 1 point, 6–25%; 2 points, 26–50%; 3 points, 51–75%; and 4 points, 76–100%) and the staining intensity score (0 points, no staining; 1 point, weak staining; 2 points, moderate staining; and 3 points, strong staining). A final IRS of > 4 indicates strong positive expression, whereas a score of < 4 indicates weak positive expression.

### Enzyme-linked immunosorbent assay

According to the instructions of an enzyme-linked immunosorbent assay (ELISA) kit (mlbio, ml202850, ml059197), 20 mg tissue was taken and added to 20 μL PBS before grinding and homogenization at 4 °C. After centrifugation at 2000 rpm for 20 min, the supernatant was taken. One hundred microliter enzyme standard reagent was added to each well, mixed with gentle shaking, covered with a plate sticker and incubated at 37 °C for 1 h. The liquid in the wells was discarded, and the wells were shaken until dry. Aliquots of 50 μL chromogenic agent A and 50 μL chromogenic agent B were added to each well, shaken gently and mixed well, and the colour was allowed to develop at 37 °C for 15 min. The measurements were zeroed against the blank wells, and the OD of the sample in each well was measured sequentially at 450 nm with an enzyme marker within 15 min of the termination of the reaction. ELISA Calc software was used to generate the standard curve and calculate the regression equation according to the concentration and OD value of the standard, and the protein contents of the samples to be tested were calculated according to the regression equation.

### Microdetermination

According to the instructions of a GSH assay kit (Hailian Biologicals, m1076450), tissues were first washed twice with PBS, and then a 0.1 g sample was weighed out. Liquid nitrogen was added, and then 1 mL reagent I was added. The mixture was ground rapidly and centrifuged at 8000 rpm for 10 min at 4 °C. Next Generate the standard curve according to instructions. Finally, in the sample tube measurements, to a 96-well plate 20 μL sample, 140 μL reagent II and 40 μL reagent III were added, mixed and left for 2 min. The absorbance at 412 nm (A2) was measured, and ΔA was defined as A2 − A1. The standard curve was generated from the standard concentrations and OD values using ELISA Calc software, and the regression equation was derived, namely, [GSH] = *y* × volume of supernatant added to the reaction system/(volume of supernatant added to the reaction system/total volume of supernatant × sample mass), from which the GSH concentration was calculated.

### Statistical processing

SPSS 25.0 software was used for statistical analysis. One-way analysis of variance was used to analyse comparisons among groups. Tukey’s test was used to compare the mean square deviations for the two groups. When the variance was not uniform, Dunnett’s T3 test was used. The data were expressed as the mean ± standard error of the mean. A *P*-value of less than 0.05 was considered to be statistically significant.

## Results

### Effect of GRg3 on the body weight of rats with GPL

**Fig. 2:**
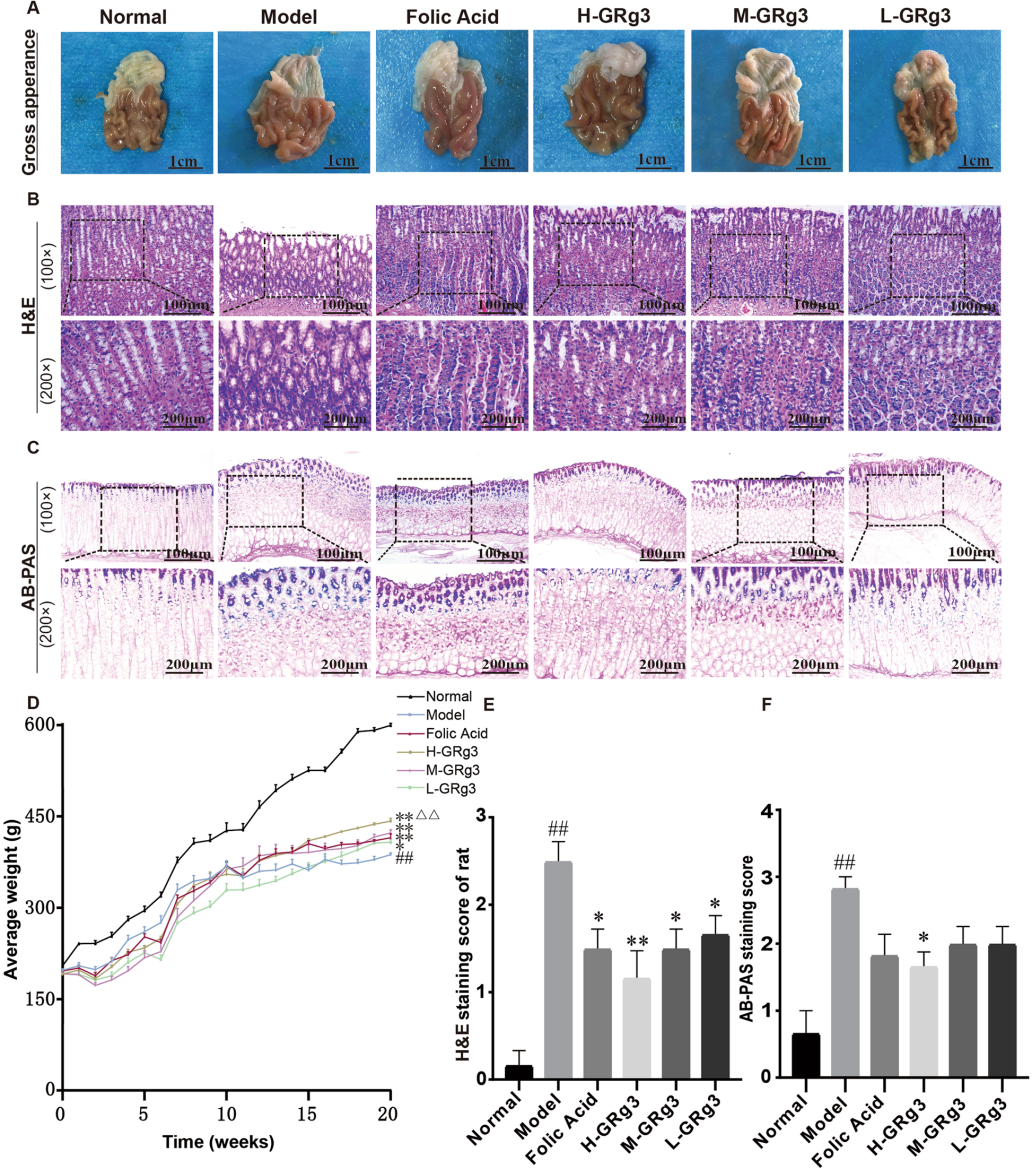
Effect of GRg3 on the body weight and histopathology of the gastric mucosa in MNNG-induced GPL rats. **A** Observations of gastric mucosa from rats with gastric precancerous lesion. **B** Histopathological changes in gastric mucosa from rats in various groups (haematoxylin and eosin staining, 100× and 200× magnification). **C** Integrity of gastric mucosa from rats in various groups (Alcian blue–periodic acid Schiff staining, 100× and 200× magnification). **D** Folded graph of body weight of rats from 0 to 20 weeks，the body weight in the 20th week results are expressed as the mean ± SEM (*n* = 10 for each group). **E** haematoxylin and eosin staining score are expressed as the mean ± SEM (*n* = 10 for each group). **F** Alcian blue–periodic acid Schiff staining score are expressed as the mean ± SEM (*n* = 10 for each group). #*P* < 0.05, ##*P* < 0.01 vs normal group; *P < 0.05, **P < 0.01 vs model group; ▲*P* < 0.05, ▲▲*P* < 0.01 vs folic acid group

As shown in (Fig. 2D), at week 20 the body weight of rats in the model group was significantly reduced in comparison with the normal group, and the difference was statistically significant (*P* < 0.01). After treatment, the body weight of rats in the folic acid and GRg3 groups was significantly higher in comparison with the model group (*P* < 0.01 and *P* < 0.05), and the high-dose GRg3 group displayed a significant increase in comparison with the folic acid group (*P* < 0.01). The above results indicated that GRg3 could effectively increase the body weight of rats with GPL.

### Effect of GRg3 on the histopathology of the gastric mucosa in rats with GPL

As shown in (Fig. 2A), in the normal group rats had smooth mucosal folds, mucus adhesion on the surface, no erosion or bleeding, uniform thickness of the gastric wall and high elasticity. In the model group rats had rough mucosal folds, low glossiness, reduced mucus adhesion on the surface, dark red tissue colour, thickened blood vessels, scattered bleeding, varying thickness of the gastric wall and reduced elasticity. After continuous treatment for 12 weeks, the gastric mucosal lesions were all alleviated to different degrees.

The degree of damage to the gastric mucosa was determined by histopathological examination. As shown in (Fig. 2B), in comparison with the normal group the model group had incomplete gastric mucosa, a less elastic gastric wall and a different basement membrane thickness. Within the disordered gastric mucosal epithelial tissue, enlarged and dilated glands were seen among the dysplastic gastric epithelial cells. In addition, the gastric mucosal epithelial cells exhibited different morphological sizes and marked heterogeneity, and inflammatory cells had infiltrated mesenchymal cells. The heterogeneous hyperplastic glands were significantly enlarged, irregularly arranged and weakly stained, which suggested a diffuse increase in abnormal heterogeneous hyperplasia in the gastric epithelium in the model group. It is noteworthy that the abnormal proliferation of gastric mucosal epithelial cells in the high-, medium- and low-dose GRg3 groups became less extensive, scattered and confined to the basement membrane side, which suggested that GRg3 had a restorative effect on the gastric mucosa in rats.

As shown in (Fig. 2B, E), The HE staining score in the model group was significantly increase in comparison with the normal group, and the difference was statistically significant (*P* < 0.01). After treatment, the HE staining score in all treatment groups was significantly lower in comparison with the model group (*P* < 0.01 and *P* < 0.05). We used AB-PAS staining to accurately determine the type of IM. As shown in (Fig. 2C), in the model group the mucinous metaplastic tissue was stained positively by AB-PAS, which indicated that the gastric fundic wall cells and principal cells were replaced by foamy cells containing neutral and acidic mucin. The gastric mucosa was extensive and stained deeply, and the substrate exhibited predominant IM. IM of incomplete cells occurred on the luminal side of the stomach, whereas IM of complete cells occurred in the lamina propria. Interestingly, the dangerous demarcation between GPL and GC, congested tubular gland structures and back-to-back tubular structures were significantly alleviated after treatment with GRg3.

As shown in (Fig. 2C, F), The AB-PAS staining score in the model group was significantly increase in comparison with the normal group, and the difference was statistically significant (*P* < 0.01). After treatment, the AB-PAS staining score in the high-dose GRg3 group was significantly lower in comparison with the model group (*P* < 0.05).

### Effect of GRg3 on apoptosis, proliferation and ROS levels in rats with GPL

In order to confirm the relationship between apoptosis and the development of GPL, we detected apoptosis by TUNEL staining. After TUNEL staining, the nuclei of normal cells were blue, whereas the nuclei of apoptotic cells were brown.

**Fig. 3:**
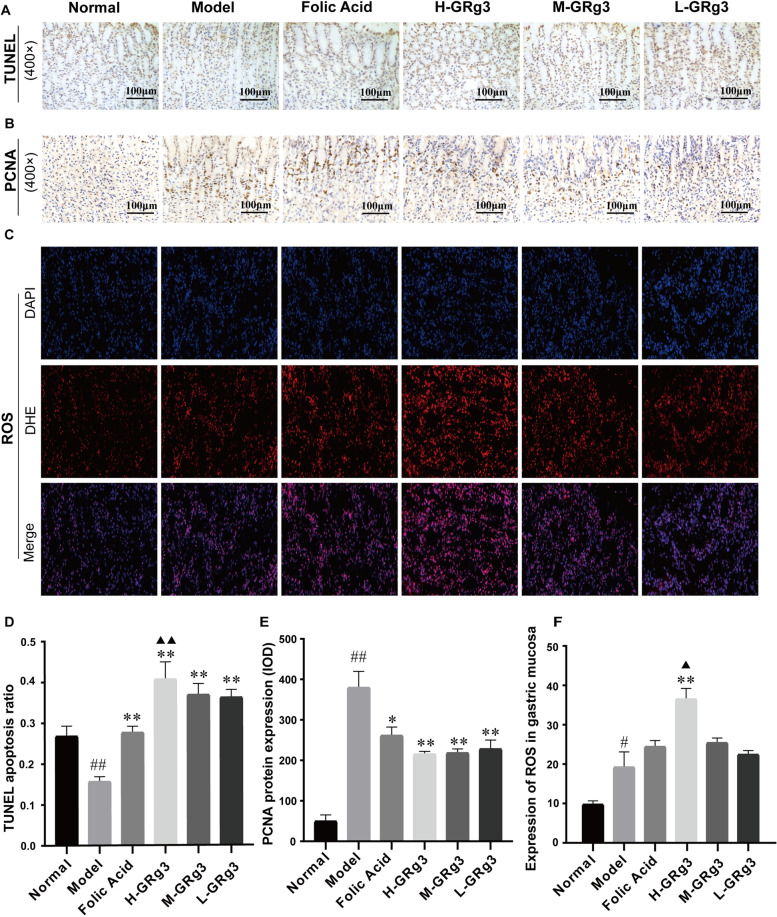
Effects of GRg3 on the production levels of apoptosis, proliferation and ROS in in MNNG-induced GPL rats. **A** Apoptosis in gastric mucosa from rats in various groups shown by terminal deoxynucleotidyl transferase dUTP nick end labelling (TUNEL) staining (400× magnification). **B** Determination of PCNA in rat gastric mucosa samples from various groups by an immunohistochemistry (IHC) assay (400× magnification) **C** Effect of GRg3 on ROS in gastric mucosa (40× magnification). **D** Apoptosis ratio shown by TUNEL staining. The results are expressed as the mean ± SEM (*n* = 10 for each group). **E** PCNA protein expression determined from cumulative mean optical density (**F**) Production of ROS in gastric mucosa. The results are expressed as the mean ± standard error of the mean (SEM) (*n* = 10 for each group). #*P* < 0.05, ##*P* < 0.01 vs normal group; *P < 0.05, **P < 0.01 vs model group; ▲*P* < 0.05, ▲▲*P* < 0.01 vs folic acid group

As shown in (Fig. 3A and D), significant apoptosis was seen in gastric mucosal epithelial cells in the normal group. Apoptosis in gastric mucosal epithelial cells in the model group was significantly reduced in comparison with the normal group, and the difference was statistically significant (*P* < 0.01). After treatment, the number of apoptotic cells in the folic acid and GRg3 groups was significantly higher in comparison with the model group (*P* < 0.01), and the high-dose GRg3 group displayed a significant increase in comparison with the folic acid group (*P* < 0.01). The above results suggest that GRg3 can effectively increase apoptosis in the gastric mucosal epithelium and inhibit excessive proliferation of gastric mucosal cells, which thus reduces the risk of cell mutation.

As shown in (Fig. 3B and E), the IHC assay demonstrated that the expression level of PCNA in gastric mucosal epithelial cells from rats, in the model group exhibited an increase in comparison with that in the normal group (*P* < 0.01). The increased expression of PCNA indicates that the proliferation of gastric mucosal epithelial cells in GPL rats is gradually out of control, which will increase the probability of deterioration of GPL to GC. After treatment, the content of PCNA in folic acid group and GRg3 group was significantly lower than that in model group (*P* < 0.01 or *P* < 0.05), which indicated that GRg3 could inhibit the proliferation of abnormal cells and delay the progression of GPL.

We believe that the production level of ROS is closely associated with apoptosis of gastric mucosal cells. We therefore used the fluorescent probe dihydroethidium to determine the production level of ROS in the epithelial tissues of the gastric mucosa. As shown in (Fig. 3C and F), ROS production was occasionally seen in the gastric mucosal tissues of rats in the normal group. ROS production in the model group exhibited a significant increase (*P* < 0.05) in comparison with the normal group. The moderate increase in ROS production could cause the accumulation of large amounts of oxidized proteins and lipids, which would increase the risk of cell mutation and promote the progression of GPL to GC. After treatment, the folic acid and GRg3 groups both displayed a increase in ROS content in comparison with the model group, and the high-dose GRg3 group displayed a significant increase in comparison with the model group (*P* < 0.01), which indicated that GRg3 could promote apoptosis inhibit abnormal cell proliferation and retard the progression of GPL by increasing the production level of ROS.

### Regulation of G6PDH, NADP and GSH contents in rats with GPL by GRg3

We believe that increases in G6PDH, NADP and GSH contents may disrupt the original redox balance in cells and thus increase the ROS concentration without triggering apoptosis, and the subsequent rapid proliferation of cells is one of the main reasons for the progression of GPL.

**Fig. 4:**
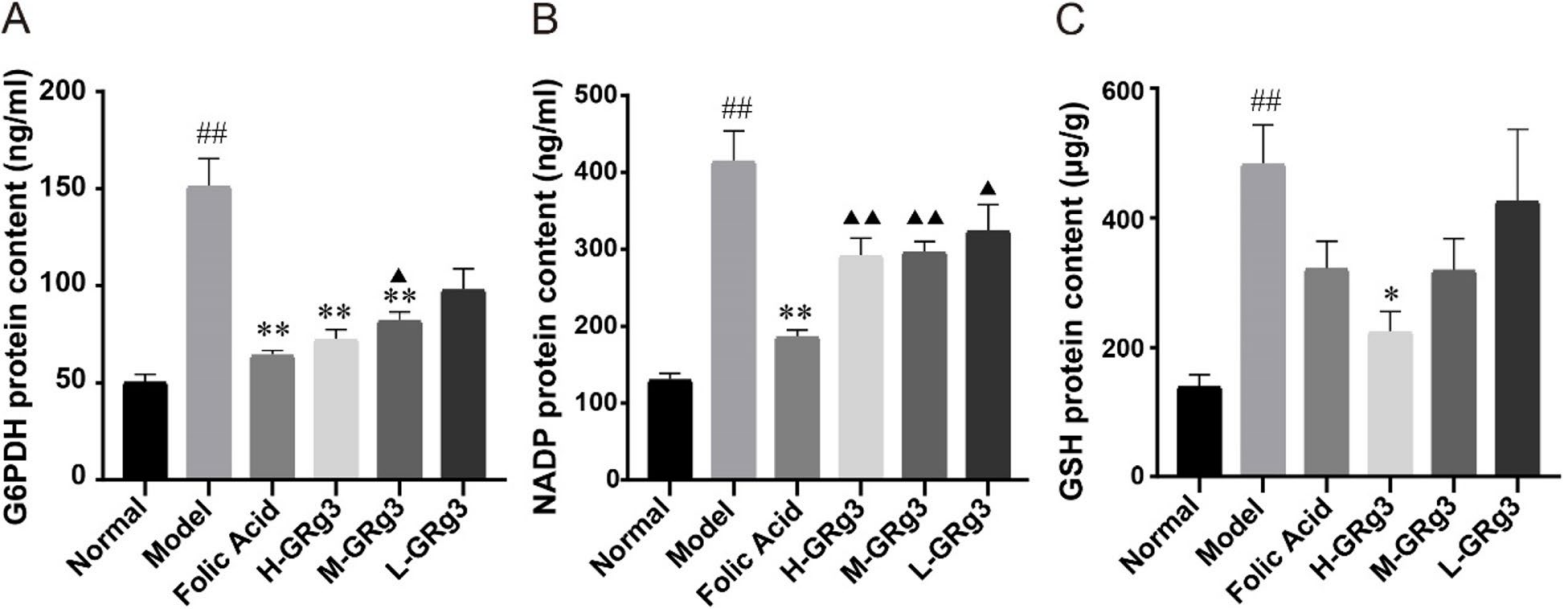
Effects of GRg3 on the production levels of G6PDH, NADP, GSH in MNNG-induced GPL rats. **A** Effect of GRg3 on production of glucose-6-phosphate dehydrogenase in gastric mucosa. **B** Effect of GRg3 on production of nicotinamide adenine dinucleotide phosphate in gastric mucosa. **C** Effect of GRg3 on production of glutathione in gastric mucosa. The results are expressed as the mean ± SEM (*n* = 10 for each group). #*P* < 0.05, ##*P* < 0.01 vs normal group; *P < 0.05, **P < 0.01 vs model group; ▲*P* < 0.05, ▲▲*P* < 0.01 vs folic acid group

G6PDH is an upstream protein of NADP that can directly affect the level of NADP. The regulatory role of the antioxidant system is particularly critical because the level of ROS in tumour cells is higher than that in normal cells owing to defects in the antioxidant enzyme system and the presence of genetic mutations and is close to the threshold limit that causes cell death. G6PDH is a key protein in the cellular antioxidant system, and therefore the study of the regulatory mechanism of G6PDH has become a current topic of research [28]. We investigated whether GRg3 acts to inhibit the progression of GPL by affecting G6PDH. As shown in (Fig. 4A), the ELISA analysis suggested that the G6PDH content appeared to have increased in the model group in comparison with the normal group (*P* < 0.01), which may indicate that the cells were affected by the upregulation of TIGAR and that the G6PDH content was thus increased. In contrast, the G6PDH contents in the folic acid group and the high- and medium-dose GRg3 groups were significantly lower in comparison with the model group (*P* < 0.01). These findings suggest that GRg3 can effectively downregulate G6PDH.

Similarly, we examined NADP, including NADP^+^ and NADPH, which are interconvertible. NADP is an important indicator of the activity and amount of G6PDH [29]. NADP is also an essential reducing agent in anabolic processes and is a key factor in the cellular scavenging of ROS. NADP protects mitochondria from damage due to ROS and is also essential for the synthesis of fatty acids, cholesterol and deoxyribonucleotides. As shown in (Fig. 4B), the model group of rats with GPL displayed a significant increase in NADP content in comparison with the normal group (*P* < 0.01), which indicated that the gastric mucosal epithelial cells were affected by TIGAR and G6PDH, which upregulated NADP. The folic acid group exhibited downregulation of NADP in comparison with the model group (*P* < 0.01). There was a downward trend in NADP content in the GRg3 groups in comparison with the model group, but it was not statistically significant, which may have been due to the small sample size. In combination with the above results, this indicated that GRg3 can reduce the level of NADP.

GSH is an antioxidant that plays the role of a free radical scavenger and detoxifying agent in cells [30]. GSH is closely associated with the development of several types of cancer and plays different roles in the early and late stages of cancer [31]. By measuring the level of GSH, we aimed to clarify the role that GSH plays in the progression of GPL to GC and to clarify whether GRg3 plays a role in the treatment of GPL by interfering with GSH.

As shown in (Fig. 4C), the GSH content in tissue from rats in the model group was significantly higher in comparison with that in the normal group (*P* < 0.01). After treatment, the GSH content in tissue from rats in each treatment group decreased to a certain extent in comparison with that in the model group and in the high-dose GRg3 groups was statistically significantly lower in comparison with that in the model group (*P* < 0.05).

### TIGAR expression is upregulated in human GPL samples in comparison with normal mucosa samples

**Fig. 5:**
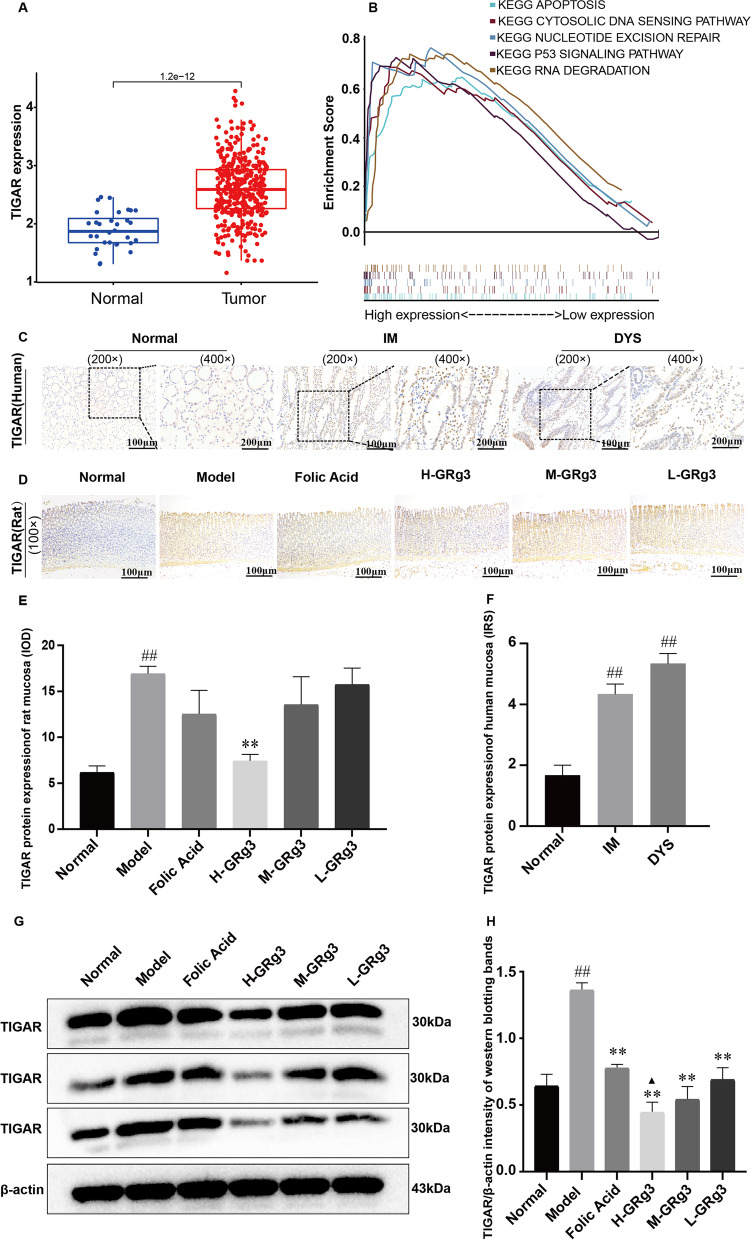
TIGAR expression is up-regulated in human GPL samples than in normal mucosa samples, and GRg3 can effectively reduce the TIGAR expression level in MNNG-induced GPL rats. **A** Expression of TP53-induced glycolysis and apoptosis regulator (TIGAR) in gastric cancer from data retrieved from the Cancer Genome Atlas database. **B** Results of enrichment analysis of a TIGAR gene set. **C** Determination of TIGAR in human gastric mucosa samples from various groups by an immunohistochemistry (IHC) assay (200× and 400× magnification). **D** Determination of TIGAR in rat gastric mucosa samples from various groups by an IHC assay (400× magnification). **E** TIGAR protein expression determined from cumulative mean optical density. The results are expressed as the mean ± SEM (*n* = 10). **F** TIGAR protein expression in human gastric mucosa determined from the immunoreactivity score. The results are expressed as the mean ± SEM (*n* = 10). **G** Determination of TIGAR in rat gastric mucosa from various groups by WB. **H** TIGAR protein expression determined by western blotting (WB). The results are expressed as the mean ± SEM (*n* = 3). #*P* < 0.05, ##*P* < 0.01 vs normal group; *P < 0.05, **P < 0.01 vs model group; ▲*P* < 0.05, ▲▲*P* < 0.01 vs folic acid group

TIGAR is a TP53-induced regulator of glycolysis and apoptosis [32, 33]. To determine the pathological role of TIGAR in the progression to GC, we investigated the expression pattern of TIGAR in GC samples using data from the TCGA database. The results are shown in (Fig. 5A), according to which TIGAR expression levels were significantly higher in GC samples in comparison with normal specimens (*P* < 0.001). This result suggests that upregulation of DLL4 may play an important role in the malignant progression of human GC.

In addition, to identify the TIGAR-associated cellular processes and signalling pathways in GC, GSEA was performed using transcriptomics data from the TCGA. The analysis indicated that upregulated TIGAR was enriched in the apoptosis, cytosolic DNA-sensing pathway, nucleotide excision repair, p53 signalling pathway and RNA degradation pathways (Fig. 5B).

We studied the expression and staining intensity of TIGAR in 52 normal gastric mucosa specimens and 69 gastric mucosa specimens from patients with GPL (see Table 1). We found that the strong positivity rate for TIGAR was 36.5% (19/52) in normal gastric mucosa specimens, whereas in GPL specimens the strong positivity rate for TIGAR was 58%, and the difference was statistically significant (*P* < 0.05). Interestingly, we found no clear correlations between TIGAR staining intensity and patients’ age and gender, the site of sampling and *Helicobacter pylori* infection (*P* > 0.05), and the difference in TIGAR staining intensity between IM and DYS was not significant (*P* > 0.05).

**Table 1:** Correlation between TIGAR positivity and clinicopathological characteristics of patients with gastric precancerous lesions

Variable	Case	Strong positivity	Weak positivity/ absent	*P*-value, strong vs weak/absent
**Age**				
< 60	44	26	18	0.803
≥ 60	25	14	11	
**Gender**				
Male	34	21	13	0.529
Female	35	19	16	
**Location of lesion**				
Body	10	5	5	0.907
Angle	13	7	6	
Antrum	34	21	13	
Multiple	12	7	5	
***Helicobacter pylori*** **infection**				
Negative	28	14	14	0.268
Positive	41	26	15	
**Histopathological category**				
Normal gastric epithelium	52	19	33	0.020
Gastric precancerous lesions	69	40	29	
Small intestinal-type metaplasia	18	10	8	0.732
Colonic-type metaplasia	16	7	9	
Mild dysplasia	15	8	7	0.409
Moderate dysplasia	12	9	3	
Severe dysplasia	8	6	2	

The semi-quantitative determination of TIGAR was performed by calculating the IRS, as shown in (Fig. 5C and F). It was found that TIGAR expression in gastric mucosa from normal subjects was at a low level, whereas after the development of GPL TIGAR expression appeared to increase. TIGAR expression in both IM and DYS specimens was significantly higher than that in specimens from normal subjects (*P* < 0.01).

### Effect of GRg3 on TIGAR expression in rats with GPL

We also examined TIGAR expression in rat gastric mucosa using both IHC and western blotting (WB) assays. As shown in (Fig. 5D and E), the IHC assay demonstrated that the expression level of TIGAR in gastric mucosal epithelial cells from rats in the model group exhibited an increase in comparison with that in the normal group (*P* < 0.01). After treatment, the expression level of TIGAR in all groups displayed different degrees of downregulation, among which that in the high-dose GRg3 group was statistically significant in comparison with that in the model group (*P* < 0.01).

Similarly, as shown in (Fig. 5G and H), the WB results confirmed the IHC assay results. The TIGAR expression level appeared to be higher in the model group in comparison with the normal group, and the difference was statistically significant (*P* < 0.01). This indicated that the fact that TIGAR regulates the cellular redox status and induces the decreased apoptosis of gastric mucosal epithelial cells is one of the main factors that lead to the development of GPL. After the intervention, the TIGAR expression level in each GRg3 group displayed a significant decrease in comparison with the model group (*P* < 0.01), which indicated that GRg3 can effectively reduce the TIGAR expression level.

In summary, GRg3 has the effect of regulating ROS, GSH, G6PDH and NADP in rats with GPL, which in turn promotes apoptosis of gastric mucosal epithelial cells, alleviates abnormal changes in the gastric mucosa and reduces the risk of progression of the disease.

## Discussion

The high mortality and morbidity rates of GC have put enormous pressure on global healthcare systems, and it is therefore important to discover potentially effective treatments for GPL. There is growing evidence that GRg3 plays a role in reducing toxic effects and enhancing the efficacy of treatment when used as an adjuvant in conventional cancer therapy [12]. Previous studies have confirmed that GRg3 can inhibit glycolysis via the PI3K/Akt/miRNA-21 pathway [34], inhibit GPL angiogenesis via downregulation of Glut1 and Glut4 [16], and promote gastric mucosal epithelial cell apoptosis via upregulation of Sp1 and downregulation of heat shock factor-1, to alleviate GPL [15]. Via this study, we found that GRg3 could also play a role in inhibiting the progression of GPL by regulating ROS and inducing apoptosis in gastric mucosal epithelial cells.

We used a rat model of GPL induced by MNNG in combination with ammonia in a composite modelling method. By staining with H&E and AB-PAS, we found that the distorted morphology, structural disorder and cellular heteromorphism in the form of epithelial glands and intestinal epithelial metaplastic lesions in the gastric mucosa of rats in the GRg3 groups were alleviated to different degrees. This indicated that GRg3 can effectively inhibit the development of IM and DYS and can effectively prevent malignant transformation of the gastric mucosa in rats with GPL.

ROS plays the role of a ‘double-edged sword’ in the development of cancer. On the one hand, in cancer cells, owing to mitochondrial dysfunction, metabolic alterations and frequent genetic mutations, the concentration of ROS increases significantly, leading to the accumulation of large amounts of oxidized proteins, DNA and lipids, which promote excessive proliferation and metastasis of cancer cells and thus play a role in the development of cancer [35]. On the other hand, when the concentration of ROS exceeds the threshold value, which triggers irreversible oxidative stress, it can promote apoptosis in tumour cells and thus hinder the development of cancer [36].

In the GPL stage, the ROS content in the model group increased significantly in comparison with the normal group, which would lead to accelerated metabolism and decreased apoptosis of gastric mucosal epithelial cells and the gradual progression of the disease. The production of NADP, G6PDH and GSH decreased after treatment, with downregulation of the upstream factor TIGAR, and the intracellular ROS content in gastric mucosal epithelial cells in each treatment group increased further in comparison with the model group. We then detected apoptosis by TUNEL staining and found that after treatment with GRg3 the apoptosis rate of gastric mucosal epithelial cells increased significantly in comparison with that of the model group, which indicated that GRg3 may induce apoptosis by regulating the production level of ROS and thus play a role in the increase inhibition of abnormal cell proliferation to retard the progression of the disease.

TIGAR is a TP53-induced regulator of glycolysis and apoptosis [32, 33]. Recent studies suggest that various cancer tissues, including those in GC, exhibit increased levels of TIGAR and that certain types of cancer are suppressed after knockdown of TIGAR [25, 37, 38].

Our experimental results suggest that TIGAR is highly expressed in GPL in both rat gastric mucosal tissues and human gastric mucosal tissues, which is consistent with the results for TIGAR expression in several types of cancer [37, 39]. However, there are no studies that indicate the role of TIGAR in GPL, and it is therefore of great interest to further investigate the exact role played by TIGAR in the progression of GPL to GC.

The present study found that TIGAR protein expression was significantly higher in the gastric mucosa of rats with GPL and human gastric mucosal epithelial cells in comparison with normal rats, which suggested that overactivation of TIGAR may be one of the main factors that promote carcinogenesis via GPL. However, after treatment with folic acid and GRg3, TIGAR protein expression in rat gastric mucosa was significantly lower in comparison with the model group. This further reduced intracellular production levels of G6PDH, NADP and GSH, which helped to increase the intracellular ROS concentration to induce apoptosis and thus served to inhibit the abnormal proliferation and differentiation of gastric mucosal epithelial cells and retard the progression of GPL to GC.

Moreover, the present study found that, although there was a decreasing trend in the NADP content after treatment with GRg3, the difference was not statistically significant, which may have been due to the small sample size. It is also possible that NADP is not a target of GRg3 in the treatment of GPL. In addition, GRg3 regulates the cellular redox status as a complex network system. Further experimental investigation is therefore needed to determine the detailed biological mechanism of the regulation of ROS by GRg3.

## Conclusion

GRg3 was effective in alleviating IM and DYS lesions and retarding the progression of the disease in rats with GPL. Our study suggests that an abnormal redox status and proliferative state of gastric mucosal epithelial cells exist during the developmental stage of GPL. Overexpression of TIGAR, NADP, GSH and G6PDH in the gastric mucosal epithelium of rats with GPL, which may be one of the causes of the increase in ROS content, is an important molecular event in the progression of GPL. After treatment with GRg3, as the expression of TIGAR, NADP, GSH and G6PDH decreased, the intracellular ROS concentration further increased, which in turn induced apoptosis and served to inhibit the abnormal proliferation and differentiation of gastric mucosal epithelial cells and retard the progression of GPL to GC. These findings provide experimental evidence for the potential use of GRg3 as a drug for the treatment of GPL. Meanwhile in future research, we can combine the pathogenesis theory [40] of traditional Chinese medicine to find more traditional Chinese medicine with a therapeutic effect on GPL.

## Data Availability

The datasets used and/or analyzed during the current study are available from the corresponding author on reasonable request. Expression of TIGAR in gastric cancer from data retrieved from the Cancer Genome Atlas database: https://portal.gdc.cancer.gov/repository?cases_offset=100&facetTab=cases&filters=%7B%22op%22%3A%22and%22%2C%22content%22%3A%5B%7B%22op%22%3A%22in%22%2C%22content%22%3A%7B%22field%22%3A%22cases.primary_site%22%2C%22value%22%3A%5B%22stomach%22%5D%7D%7D%2C%7B%22op%22%3A%22in%22%2C%22content%22%3A%7B%22field%22%3A%22cases.project.program.name%22%2C%22value%22%3A%5B%22TCGA%22%5D%7D%7D%2C%7B%22op%22%3A%22in%22%2C%22content%22%3A%7B%22field%22%3A%22cases.project.project_id%22%2C%22value%22%3A%5B%22TCGA-STAD%22%5D%7D%7D%5D%7D&searchTableTab=cases, and Gene Expression Omnibus database: https://www.ncbi.nlm.nih.gov/geo/query/acc.cgi?acc=GSE54129

## Abbreviations

GRg3: Ginsenoside Rg3
GPL: Gastric precancerous lesion
MNNG: N-methyl-N′-nitro-N-nitrosoguanidine
ROS: Reactive oxygen species
TIGAR: TP53-induced glycolysis and apoptosis regulator
NADP: Nicotinamide adenine dinucleotide phosphate
G6PDH: Glucose-6-phosphate dehydrogenase
GSH: Glutathione
IM: Intestinal metaplasia
DYS: Gastric epithelial dysplasia
GC: Gastric cancer
PI3K: P-phosphoinositide 3-kinase
H&E: Haematoxylin and eosin
AB-PAS: Alcian blue–periodic acid Schiff
TUNEL: Transferase dUTP nick end labelling
PBS: Phosphate-buffered saline
HRP: Streptavidin–horseradish peroxidase
TBST: Tris-buffered saline–Tween-20
TCGA: The Cancer Genome Atlas
GSEA: Gene set enrichment analysis
IRS: Immunoreactivity score
IHC: Immunohistochemical
ELISA: Enzyme-linked immunosorbent assay
WB: Western blotting
