# Correction: Ginsenoside Rg3 induces apoptosis and inhibits proliferation by down-regulating TIGAR in rats with gastric precancerous lesions

**DOI:** 10.1186/s12906-023-03882-4

**Published:** 2023-02-27

**Authors:** Shangbin Lv, Xiaodong Chen, Yu Chen, Daoyin Gong, Gang Mao, Caifei Shen, Ting Xia, Jing Cheng, Zhaoliang Luo, Yu Cheng, Weihong Li, Jinhao Zeng

**Affiliations:** 1 Basic Medical College, Chengdu University of Traditional Chinese Medicine, Chengdu, China; 2 Department of Gastrointestinal Surgery, Sichuan Cancer Hospital, School of Medicine, University of Electronic Science and Technology of China, Chengdu, China; 3 Hospital of Chengdu University of Traditional Chinese Medicine, Chengdu, China; 4 Digestive Endoscopy Center, Hospital of Chengdu University of Traditional Chinese Medicine, Chengdu, China; 5 Chengdu University of Traditional Chinese Medicine, Chengdu, China; 6 Sichuan University West China Hospital Ganzi Hospital, Ganzi, China; 7 Department of Chinese Internal Medicine, Hospital of Chengdu University of Traditional Chinese Medicine, Chengdu, China; 8 TCM Regulating Metabolic Diseases Key Laboratory of Sichuan Province, Hospital of Chengdu University of Traditional Chinese Medicine, Chengdu, China

**Correction: BMC Complement Med Ther 22, 188 (2022)**

**https://doi.org/10.1186/s12906-022-03669-z**

Following publication of the original article [1], the authors identified an error in Figs. 3 and 5. The correct figure and explanation of the corrections are given below.

In Fig. 3 the following were corrected:

1. We found that pictures of Folic acid, M-Grg3 and L-Grg3 in Fig. 3 were misused. We re-selected the typically representative images of ROS in Fig. 3C, and the selected images were more representative. This operation does not affect the scientific conclusion.
2. The blue channel is DAPI and the red channel is DHE. And the DAPI and DHE were mis-labeled with each other in the present Fig. 3. We thus have re-labeled them in the right place in new corrected Fig. 3.

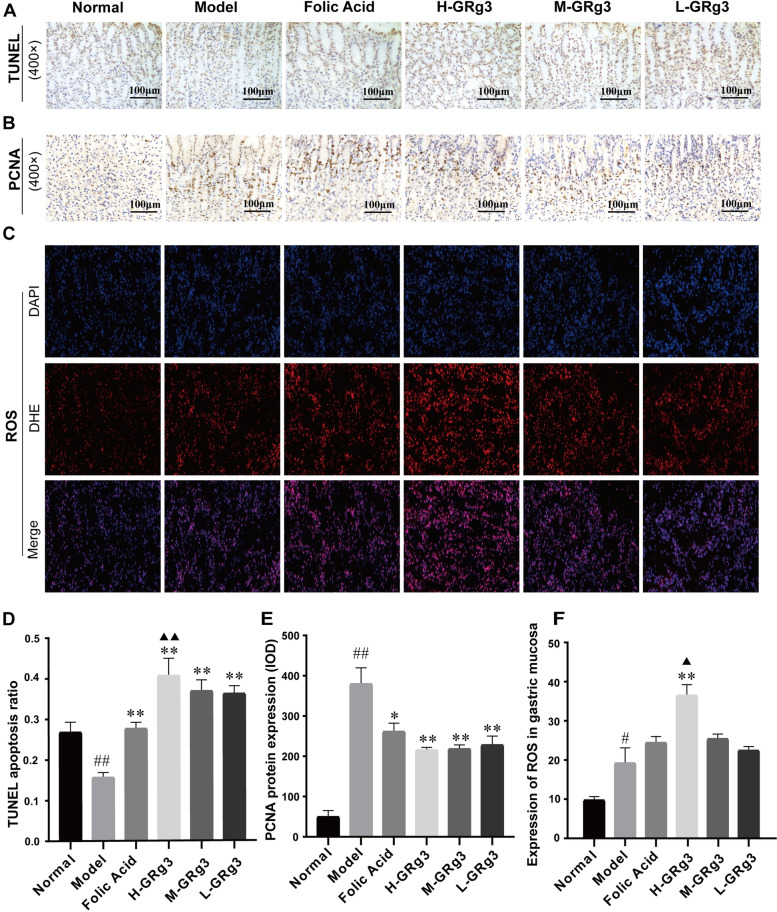

Also the following corrections were made to Figure 5:

1. The magnification of Fig. 5D should be 400× instead of 100×
2. After careful checking for full raw data and recalculating the data, we found that *P* value label should be “##” or “**” instead of “#” or “*” in Fig. 5E and 5F. This operation did not change the scientific conclusion.
3. The word “noramal” was corrected to “normal”.

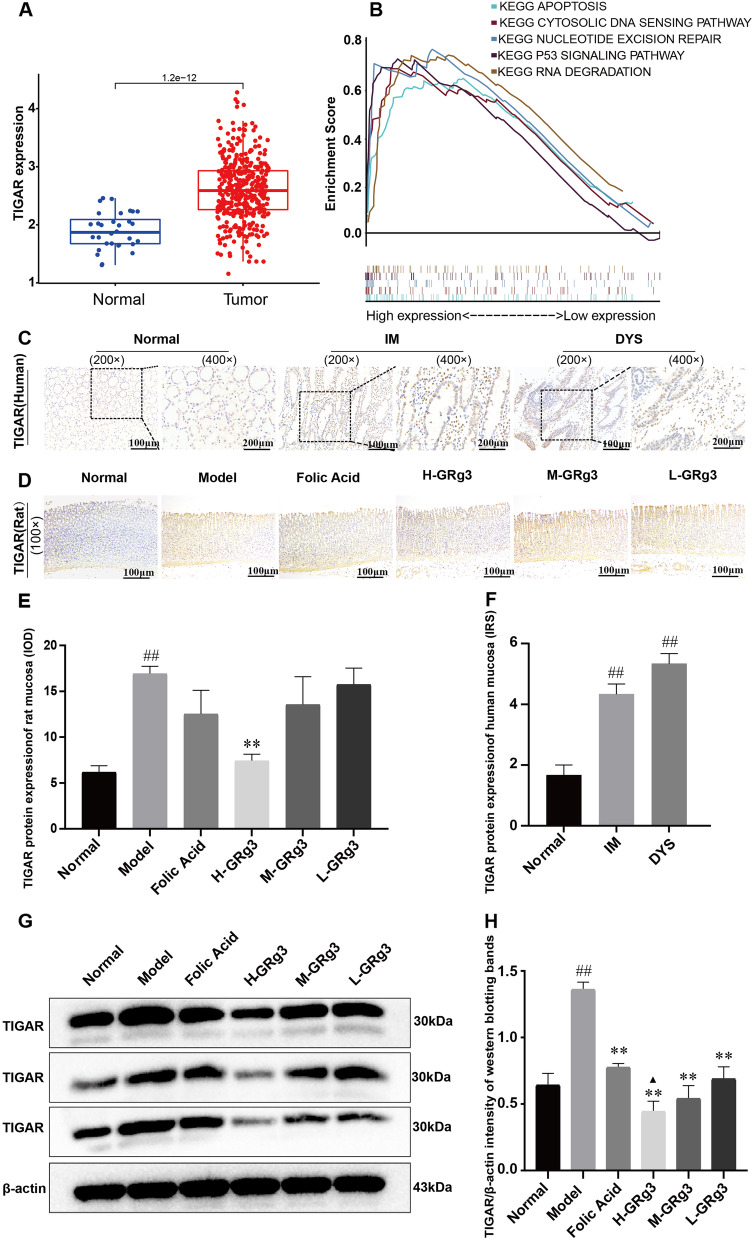

The original article has been corrected.
